# Genetic variability in sodium-glucose cotransporter 2 and glucagon-like peptide 1 receptor effect on glycemic and pressure control in type 2 diabetes patients treated with SGLT2 inhibitors and GLP-1RA in the everyday clinical practice

**DOI:** 10.3389/fendo.2025.1547920

**Published:** 2025-07-07

**Authors:** Gašper Tonin, Katja Goričar, Tanja Blagus, Andrej Janež, Vita Dolžan, Jasna Klen

**Affiliations:** ^1^ Faculty of Medicine, University of Ljubljana, Ljubljana, Slovenia; ^2^ Faculty of Arts, University of Ljubljana, Ljubljana, Slovenia; ^3^ Pharmacogenetics Laboratory, Institute of Biochemistry and Molecular Genetics, Faculty of Medicine, University of Ljubljana, Ljubljana, Slovenia; ^4^ Department of Internal Medicine, Faculty of Medicine, University of Ljubljana, Ljubljana, Slovenia; ^5^ Division of Internal Medicine, Department of Endocrinology, Diabetes and Metabolic Diseases, University Medical Centre Ljubljana, Ljubljana, Slovenia; ^6^ Division of Surgery, Department of Abdominal Surgery, University Medical Centre Ljubljana, Ljubljana, Slovenia

**Keywords:** polymorphism, SLC5A2, GLP1R, treatment response, type 2 diabetes mellitus, SGLT2, GLP-1RA, everyday clinical practice

## Abstract

**Aim:**

We investigated the impact of genetic polymorphisms in the *GLP1R* and *SLC5A2* genes on the response to treatment with glucagon-like peptide-1 receptor (GLP-1R) agonists and sodium-glucose co-transporter-2 (SLGT2) inhibitors in patients with type 2 diabetes mellitus (T2DM) in everyday clinical practice.

**Methods:**

In our prospective interventional cohort open-label real-world genetic association study (DRKS-ID: DRKS00034478, https://drks.de/search/en/trial/DRKS00034478), we enrolled 161 clinically well-defined T2DM patients who received SGLT2 inhibitors and/or GLP-1R agonists alongside other medications for 3–6 months. The study’s primary outcomes (HbA1c, body mass, and blood pressure) were measured before the treatment and at the follow-up at 3–6 months. *GLP1R* rs6923761, rs10305420, and *SLC5A2* rs9934336 genotypes were determined by competitive allele-specific polymerase chain reaction. In patients receiving GLP-1R agonists, we analyzed the effect of *GLP1R* polymorphisms on the patients’ response to treatment, while in patients receiving SGLT2 inhibitors, we analyzed the impact of the *SLC5A2* polymorphism on the treatment effect.

**Results:**

Treatment with prescribed antihyperglycemic drugs improved all primary outcomes (p < 0.050). The normal *GLP1R* rs6923761 G allele was associated with a greater reduction in HbA1c with GLP-1R agonists treatment than the polymorphic A allele in the dominant model (p = 0.029).

**Conclusions:**

The prevalent polymorphic A allele of *GLP1R* rs6923761 polymorphism was associated with the clinically relevant lower glycemic response to GLP-1R agonists. The described impact extends to everyday clinical practice, indicating that knowledge of these genetic polymorphisms could facilitate the development of targeted and personalized therapy in managing T2DM.

## 1 Introduction

As type 2 diabetes mellitus (T2DM) continues to rise in prevalence, there is increasing interest in investigating its genetic, epigenetic, and transcriptomic foundations. In recent years, T2DM treatment guidelines have emphasized a holistic approach, including body mass reduction, control of cardiovascular risk factors, associated diseases, and cardio-renal protection, in addition to glycemic control with medication. This comprehensive strategy is crucial for preventing late T2DM complications and increasing patients’ quality of life (1, 2). In the context of personalized medicine, there is growing significance in tailoring the optimal treatment for individuals based on different biomarkers, including their genotype. This approach might protect patients from unnecessary treatments, enhance therapeutic efficacy, and promote cost-effectiveness within the healthcare system. Consequently, the utilization of pharmacogenomics stands out as one of the main pathways toward personalized diabetology (3).

Glucagon-like peptide-1 (GLP-1) receptor agonists (GLP-1RA) are a newer class of antihyperglycemic drugs with various effects. They act by improving glucose-dependent insulin secretion, inhibiting glucagon secretion, reducing hepatic gluconeogenesis, slowing gastric emptying, increasing peripheral tissue responsiveness, and enhancing satiety via their effect on the central nervous system (4). GLP-1R is encoded by the *GLP1R* gene, located on chromosome 6p21.2 (5, 6). Alterations in this gene are associated with a change in its function, affecting both its physiological function and its response to GLP-1RA. Several different changes can affect the genetic variability of *GLP1R*, especially changes in regulatory regions (e.g. promoter, 3’-untranslated region) and coding regions (exons and exon-intron boundaries) (5, 6). The most common single nucleotide polymorphisms (SNP) leading to changes in GLP-1R function are rs1042044 (p.Leu260Phe, A>C), rs10305420 (p.Pro7Leu, C>T), rs6923761 (p.Gly168Ser, G>A), and rs3765467 (p.Arg131Gln, G>A) (7). These polymorphisms were shown to lead to changes in cell surface receptor expression, ligand binding, and cAMP mediated intracellular signaling (8).

Sodium-Glucose Co-Transporter-2 (SGLT2) inhibitors are antihyperglycemic drugs that inhibit glucose reabsorption in the proximal tubule via SGLT2, resulting in glucosuria, which in turn reduces hyperglycemia (9). As SGLT2 is also found in pancreatic α-cells, it has been hypothesized that SGLT2 inhibitors might differentially affect glucagon secretion, but this has not yet been experimentally confirmed (10). The SGLT2 protein is transcribed by the gene *SLC5A2* (solute carrier family 5 member 2), located on chromosome 16 (11, 12). Although some studies have already shown that genetic changes in this gene could influence the development of T2DM, its late complications, and the response to treatment with SGLT2 inhibitors, results are not consistent (9). The rs9934336 polymorphism is located in intron 1 of the *SLC5A2* gene, but its precise molecular role is not yet fully understood (13).

Pharmacogenomic mechanisms and findings from genome-wide association studies (GWAS) suggest that genetic polymorphisms may significantly influence patients’ response to therapy (14–16). Despite being common in the population, their association with treatment response in T2DM patients is still poorly understood. In this study, we aimed to elucidate the association between gene polymorphisms related to two pharmacological targets of emerging antihyperglycemic medications - GLP-1RAs and SGLT2 inhibitors - and the clinical response of patients undergoing treatment with these drugs in everyday clinical practice.

## 2 Materials and methods

We conducted a prospective interventional cohort open-label genetic association study in an everyday clinical setting, investigating the impact of *GLP1R* and *SLC5A2* polymorphisms on the response to 3–6 months of treatment with GLP-1RAs and SGLT2 inhibitors in patients with T2DM.

The study was approved by the National Ethics Committee (0120-209/2018/8) and conducted in accordance with the Declaration of Helsinki. All patients were informed of the purpose and conduct of the study and gave written informed consent to participate in the study.

The trial was registered in the German Clinical Trials Register (DRKS-ID: DRKS00034478).

### 2.1 Patients

The clinical part of the study was carried out at the University Medical Centre Ljubljana and in the diabetes outpatient clinic of Health Centre Kočevje. The molecular genetic analysis was conducted at the Institute of Biochemistry and Molecular Genetics, Faculty of Medicine, University of Ljubljana.

Patients were included if they had previously been diagnosed with T2DM and met the national clinical guidelines inclusion criteria for treatment with antihyperglycemic drugs (SGLT2 inhibitors and/or GLP-1RA inhibitors), and had been therefore prescribed the corresponding drug (17–19). Patients were enrolled only if they gave written informed consent to participate and were counseled on the use and side effects of the antihyperglycemic drugs introduced. We excluded all patients with other types of diabetes mellitus and patients who could be expected to have poorer compliance or a significant impact of other factors on treatment (patients with significant cognitive impairment or psychiatric disorder, patients abusing alcohol or other drugs, patients with cancer or a history of cancer in the last 5 years).

### 2.2 Study design and outcomes

All patients were prescribed newer antihyperglycemic drugs at the start of the study. However, they were also allowed to receive other medicines at the same time (**Supplementary Table 1**). Among GLP-1Ras, patients were prescribed either semaglutide, dulaglutide or liraglutide.

From the patient’s medical records and medical history, we obtained information on the duration of T2DM, previous T2DM treatment, the presence of hyperlipidemia, arterial hypertension, late complications of T2DM, and smoking. Additionally, all patients underwent a physical examination; blood pressure, body height, and body mass were measured. In all patients, glycated hemoglobin (HbA1c), glucose, lipid profile, urea, creatinine, estimated glomerular filtration rate (eGFR), and liver enzymes were also determined from fasting blood samples.

The primary outcome variables were HbA1c, body mass, and blood pressure. The reassessment occurred 3–6 months later at the follow-up visit, which was scheduled based on the physician’s clinical judgment, considering the patient’s presentation.

Patients also had peripheral blood or buccal swab samples taken for DNA isolation and genetic analysis. As the patients were receiving combinations of different antihyperglycemic medications, subgroup analyses were conducted for those receiving GLP-1RA or SGLT2 inhibitors, respectively. In the first subgroup, we assessed the effect of the two *GLP1R* polymorphisms, while in the second subgroup, we examined the influence of the *SLC5A2* polymorphism on the treatment response.

### 2.3 SNP selection, DNA isolation, and genotyping process

In this study, we investigated two *GLP1R* polymorphisms (rs6923761 and rs10305420) and one *SLC5A2* polymorphism (rs9934336). We included putatively functional polymorphisms with high minor allele frequency (MAF) in the European population, previously described in the literature.

DNA isolation was performed according to the instructions of the manufacturer of E.Z.N.A.^®^ SQ II Blood DNA Kit (Omega Bio-tek, Inc., USA) and the QIAamp DNA Mini Kit (Qiagen, Germany). All three genotypes were determined using competitive allele-specific polymerase chain reaction (KASP) using KASP™ assays and KASP-TF Master mix (LGC Bioresearch Technologies, UK) according to the manufacturer’s instructions. Polymerase chain reactions (PCR) were performed in a ProFlex PCR System (Applied Biosystems, USA) and the fluorescent dye signal was measured using a FLUOstar Omega Microplate Reader (BMG Labtech, Germany).

### 2.4 Statistical analysis

Descriptive statistics were used to describe the sample, with continuous variables represented by the median and interquartile range (25th to 75th percentiles), and categorical variables presented as frequencies. The normality of the distribution of the continuous variables was assessed using the Shapiro-Wilk test. As the values were not normally distributed, non-parametric statistical tests were employed: the Mann-Whitney test for two independent samples and the Wilcoxon test for related samples (comparing data from the same patient at different time points). All tests were two-tailed, and corresponding statistical tests are indicated alongside the tables presenting the results. Moreover, for each polymorphism, we calculated genotype frequencies and MAF. To assess the agreement of the genotype frequencies with the Hardy-Weinberg equilibrium (HWE), we used the χ^2^ test. The dominant genetic model was used in all analyses.

Statistical analysis was performed using IBM SPSS Statistics 27.0 (IBM Corporation, Armonk, NY, USA). Missing data are addressed in the corresponding tables. The level of statistical significance was set to 0.05.

## 3 Results

### 3.1 Patient characteristics

In total, 161 T2DM patients were included in the final analysis. According to the guidelines for the treatment of T2DM, patients received several antihyperglycemic drugs concomitantly (**Supplementary Table 1**). The most common set of drugs received by patients was a combination of an SGLT2 inhibitor, biguanides, and insulin (N = 24).

The clinical and laboratory characteristics of the patients before starting treatment with SGLT2 inhibitors or GLP-1RAs are presented in **Table 1**. A higher proportion of patients had microvascular complications (28.0%) than macrovascular complications (13.0%). Retinopathy was the most frequent microvascular complication (21.7%) and ischemic heart disease was the most frequent macrovascular complication (10.6%). The prevalence of the micro- and macrovascular late complications of T2DM in our sample is shown in **Supplementary Table 2**.

**Table 1:** Pretreatment clinical and laboratory characteristics of all patients (N = 161), patients in a GLP-1RA subgroup (N = 70), and patients in a SGLT2 inhibitors subgroup (N = 124).

Variable [number of patients without data]	Category/unit	N (%)/median (25%-75%)		
		Whole group	GLP-1RA group	SGLT2 inhibitors group
Sex	Men	101 (62.7)	40 (57.1)	82 (66.1)
	Women	60 (37.3)	30 (42.9)	42 (33.9)
Age	Years	69 (61-75.5)	66 (59.8-73)	69 (62-75.8)
Duration of T2DM	Years	16 (10-23.5)	15 (9.5-22)	16 (10-24)
Smoking	No	76 (47.2)	35 (50)	54 (43.5)
	Yes	85 (52.8)	35 (50)	70 (56.5)
BMI [1]	kg/m^2^	31.6 (28.0-35.5)	33 (29-38)	30 (27-34)
Fasting glucose [2]	mmol/l	8.0 (6.7-10.0)	8.2 (6.8-10.4)	7.8 (6.7-9.9)
Total cholesterol [3]	mmol/l	4.2 (3.4-4.8)	4.1 (3.4-4.8)	4.3 (3.4-4.9)
HDL-cholesterol [4]	mmol/l	1.1 (0.9-1.3)	1.0 (0.9-1.2)	1.1 (0.9-1.3)
LDL-cholesterol [4]	mmol/l	2.2 (1.7-2.9)	2.3 (1.7-2.9)	2.2 (1.7-2.9)
TAG [3]	mmol/l	1.8 (1.3-2.6)	1.8 (1.2-2.8)	2.0 (1.3-2.7)
eGFR [4]	ml/min/1.73 m^2^	79 (66.8-90)	82 (69.5-90)	78.5 (66.5-90)
AST [3]	µkat/l	0.39 (0.30-0.48)	0.39 (0.30-0.48)	0.37 (0.30-0.48)
ALT [3]	µkat/l	0.50 (0.38-0.65)	0.51 (0.39-0.66)	0.49 (0.36-0.66)
γ-GT [3]	µkat/l	0.38 (0.26-0.57)	0.38 (0.26-0.53)	0.38 (0.27-0.58)
Total bilirubin [8]	µmol/l	10 (8-13)	11 (8-14)	10 (7-13)
Direct bilirubin [34]	µmol/l	3 (2-4)	3 (2-4)	3 (2-4)
Diagnosed dyslipidemia	No	48 (29.8)	24 (34.3)	31 (25)
	Yes	113 (70.2)	46 (65.7)	93 (75)
Statin treatment [3]	No	47 (29.7)	23 (33.3)	33 (27)
	Yes	111 (70.3)	46 (66.7)	89 (73)
Arterial hypertension	No	2 (1.2)	1 (1.4)	1 (0.8)
	Yes	159 (98.8)	69 (98.6)	123 (99.2)

Categorical variables are presented as N (%), continuous variables as median, and interquartile range (25th and 75th percentile). N, number of patients; T2DM, type 2 diabetes mellitus; BMI, body mass index; HDL, high-density lipoprotein. HDL, high-density lipoprotein; LDL, low-density lipoprotein; TAG, triacylglycerides; eGFR, estimated glomerular filtration rate; AST, aspartate aminotransferase; ALT, alanine aminotransferase; γ-GT, γ-glutamyl transferase.

**Figure 1:**
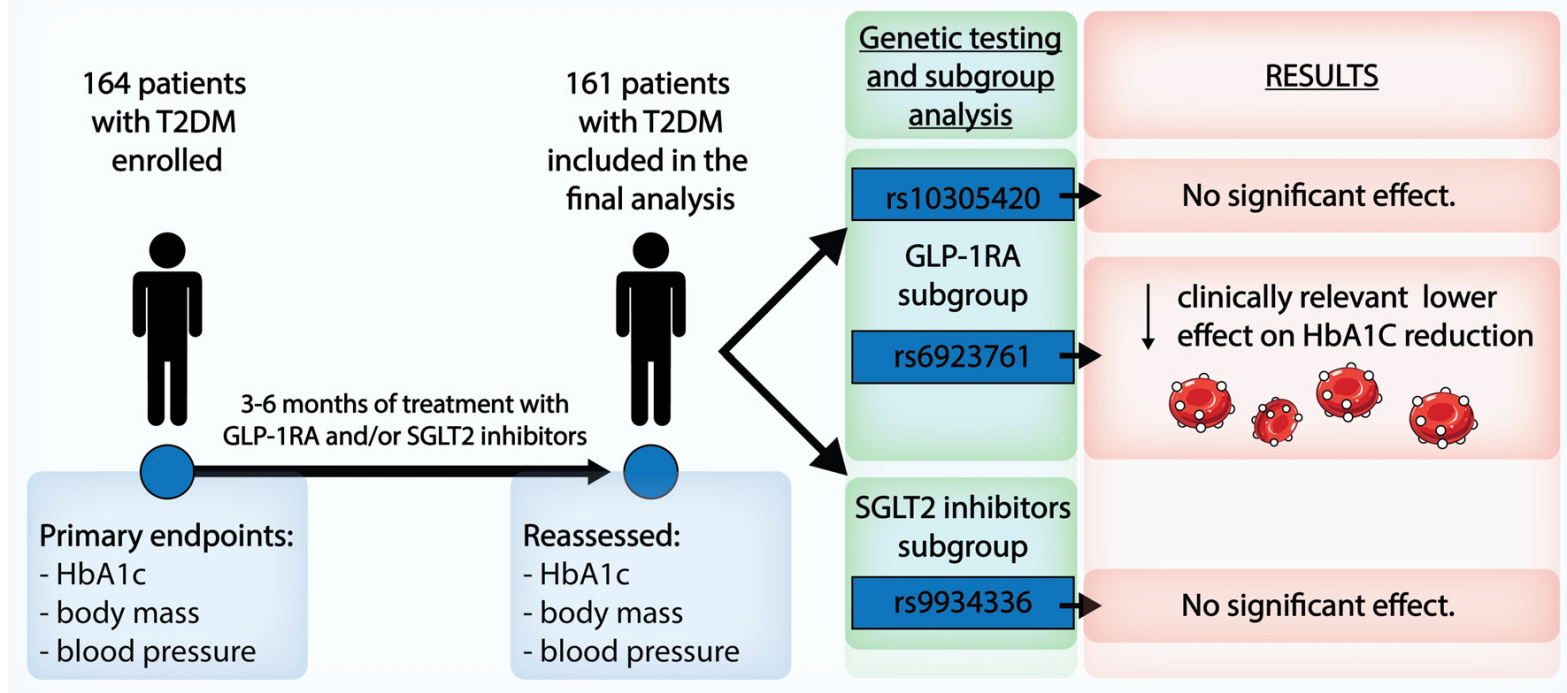
Research workflow and main results showing that *GLP1R* rs6923761 polymorphic allele correlates with lower HbA1c reduction with GLP-1RA.

In the whole group of patients, HbA1c (p < 0.001), body mass (p < 0.001), systolic blood pressure (p < 0.001), and diastolic blood pressure (p = 0.037) were statistically significantly reduced following the prescribed treatment (**Supplementary Table 3**). Study flowchart and main results are presented in **Figure 1**.

### 3.2 Results of molecular genetic analysis

The genotype distribution of selected polymorphisms in the whole group is shown in **Table 2**. All frequencies of the genotypes studied were consistent with HWE (p-value > 0.05 for all).

**Table 2:** Frequency distribution of the investigated polymorphisms.

SNP	Genetic change	Genotype	N (%)	MAF (%)	pHWE
*GLP1R* rs10305420 [4]	c.20C>T (p.Pro7Leu)	CC	77 (49.0)	30.6	0.618
		CT	64 (40.8)		
		TT	16 (10.2)		
*GLP1R* rs6923761	c.502G>C (p.Gly168Ser)	GG	80 (49.7)	31.4	0.128
		GA	61 (37.9)		
		AA	20 (12.4)		
*SLC5A2* rs9934336 [1]	g.6435G>A	GG	87 (54.4)	26.9	0.561
		GA	60 (37.5)		
		AA	13 (8.1)		

[], number of patients in whom we were unable to determine the genotype; GLP1R, glucagon-like peptide 1 receptor gene; SLC5A2, solute carrier family 5 member 2; Pro, proline; Leu, leucine; Gly, glycine; Ser, serine; A, adenine; G, guanine; C, cytosine; T, thymine; MAF, minor allele frequency; pHWE, p-value of the χ^2^ test for Hardy-Weinberg equilibrium.

### 3.3 Effect of *GLP1R* polymorphisms on treatment response to GLP-1RA

In the group receiving GLP-1RAs (N = 70), semaglutide was the most often prescribed (N = 37; 52.9%), followed by dulaglutide (N = 19; 27.1%) and liraglutide (N = 14, 20.0%). GLP-1RAs demonstrated a statistically significant effect on reducing all observed outcome variables across the entire group (all p < 0.05, **Supplementary Table 4**). The effect of *GLP1R* polymorphisms on the change in HbA1c, body mass, and blood pressure after treatment with GLP-1RAs is shown in **Table 3**.

**Table 3:** Impact of *GLP1R* polymorphisms (rs10305420 and rs6923761) on changes in the studied outcomes: HbA1c, body mass, and blood pressure.

HbA1c			
SNP	Genotype	Change in HbA1c (%) median (25%-75%)	p-value^a^
*GLP1R* rs10305420	CC	-1.1 (-1.8 to -0.5)	0.397
	CT+TT	-1 (-1.9 to 0.2)	
*GLP1R* rs6923761	GG	-1.4 (-2.4 to -0.5)	**0.029**
	GA+AA	-0.8 (-1.4 to -0.1)	
Body mass			
SNP	Genotype	Body mass change (kg) median (25%-75%)	p-value
*GLP1R* rs10305420	CC	-4.5 (-7 to -1)	0.856
	CT+TT	-3 (-7 to -1)	
*GLP1R* rs6923761	GG	-4 (-7 to -1)	0.826
	GA+AA	-4 (-7 to -1)	
Systolic blood pressure			
SNP	Genotype	Change in systolic blood pressure (mmHg) median (25%-75%)	p-value
*GLP1R* rs10305420	CC	-8 (-18.8 to 5)	0.497
	CT+TT	-10 (-20 to 1.5)	
*GLP1R* rs6923761	GG	-9 (-18 to 5)	0.372
	GA+AA	-10 (-20 to 0)	
Diastolic blood pressure			
SNP	Genotype	Change in diastolic blood pressure (mmHg) median (25%-75%)	p-value
*GLP1R* rs10305420	CC	-5 (-9.5 to 5)	0.512
	CT+TT	-1 (-5 to 3.5)	
*GLP1R* rs6923761	GG	-5 (-8 to 1)	0.268
	GA+AA	-1 (-7 to 5)	

a To calculate p-values, we used the Mann-Whitney test. HbA1c, hemoglobin A1c, also glycated hemoglobin; A, adenine; G, guanine; C, cytosine; T, thymine.

**Figure 2:**
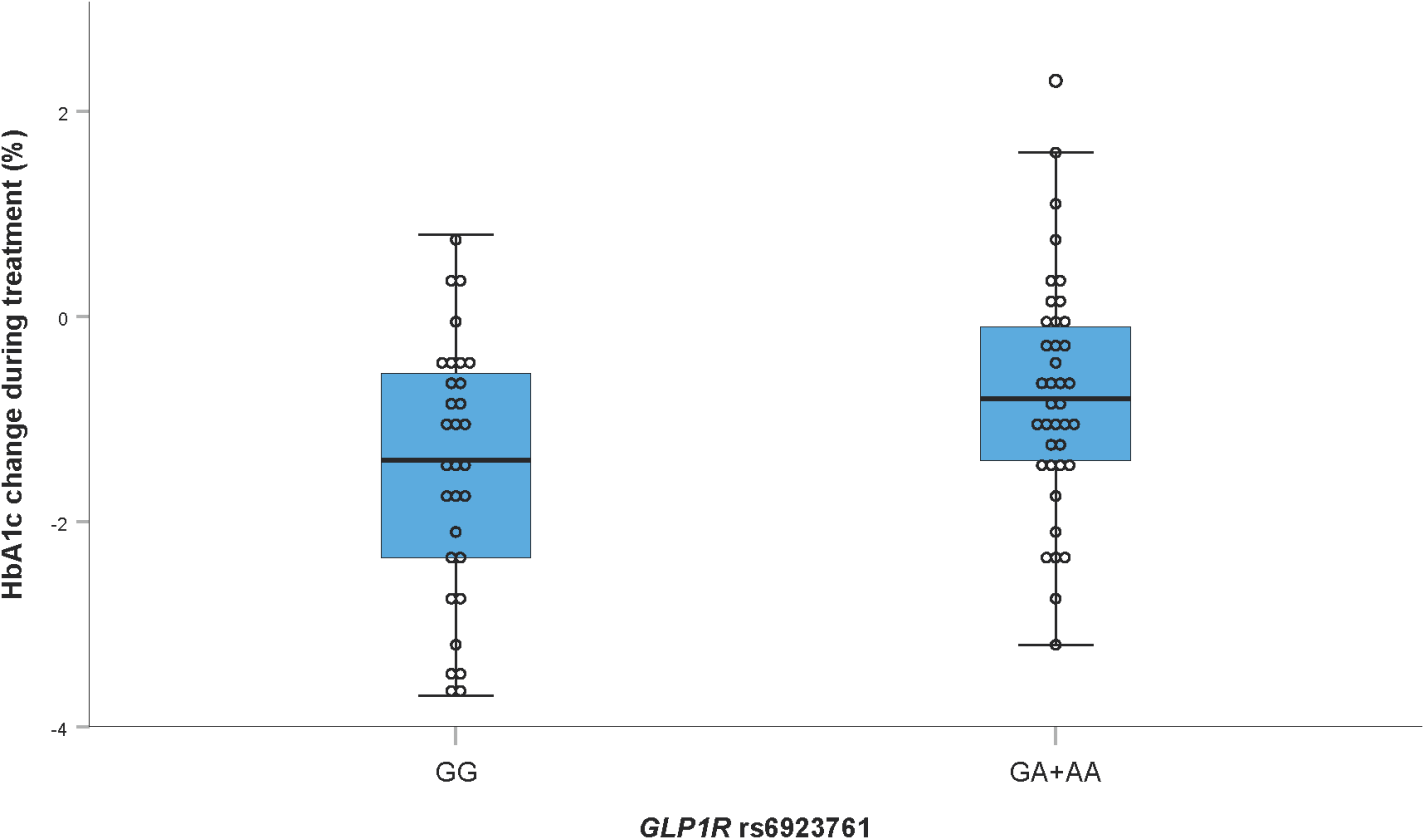
Effect of *GLP1R* rs6923761 genotype on HbA1c change following GLP-1RA treatment.

As shown in **Figure 2**, the normal *GLP1R* rs6923761 G allele was statistically significantly associated with a greater reduction in HbA1c than the polymorphic A allele in the dominant genetic model (p = 0.029). Chosen *GLP1R* polymorphisms were not significantly associated with body mass or blood pressure changes upon treatment.

### 3.4 Effect of *SLC5A2* genetic polymorphisms on treatment response to SGLT2 inhibitors

A total of 124 patients received SGLT2 inhibitors. Among these medications, patients most frequently received empagliflozin (N = 65) and slightly less frequently dapagliflozin (N = 59). In this group, SGLT2 inhibitors reduced HbA1c (p < 0.001), body mass (p < 0.001), and systolic blood pressure (p = 0.020), but not diastolic blood pressure (p = 0.131) (**Supplementary Table 5**).

The effect of the *SLC5A2* rs9934336 polymorphism on the change of the primary outcome variables (HbA1c, body mass, and blood pressure) is shown in **Table 4**. No statistically significant associations between genotype and treatment response were observed for the investigated *SLC5A2* polymorphism.

**Table 4:** Impact of *SLC5A2* rs9934336 on changes in the studied outcomes: HbA1c, body mass, and blood pressure.

HbA1c			
SNP	Genotype	Change in HbA1c (%) median (25%-75%)	p-value^a^
*SLC5A2* rs9934336	GG	-0.8 (-1.6 to 0.1)	0.567
	GA+AA	-0.5 (-1.5 to 0)	
Body mass			
SNP	Genotype	Body mass change (kg) median (25%-75%)	p-value
*SLC5A2* rs9934336	GG	-3 (-5 to -1)	0.752
	GA+AA	-2.5 (-5.8 to -1)	
Systolic blood pressure			
SNP	Genotype	Change in systolic blood pressure (mmHg) median (25%-75%)	p-value
*SLC5A2* rs9934336	GG	0 (-16.8 to 9.8)	0.403
	GA+AA	-3 (-15 to 4.5)	
Diastolic blood pressure			
SNP	Genotype	Change in diastolic blood pressure (mmHg) median (25%-75%)	p-value
*SLC5A2* rs9934336	GG	0 (-6.8 to 5)	0.600
	GA+AA	-2 (-9.8 to 5)	

a To calculate p-values, we used the Mann-Whitney test. *SLC5A2*, *solute carrier family 5 member 2*; HbA1c, hemoglobin A1c, *also* glycated hemoglobin; A, adenine; G, guanine.

## 4 Discussion

We investigated the impact of genetic factors on the clinical response to treatment with SGLT2 inhibitors and GLP-1RAs in clinically well-described patients with T2DM. In our study, we have shown that the carriers of the *GLP1R* rs6923761 normal allele responded with a significantly greater reduction in HbA1c than those with the polymorphic allele when treated with GLP-1RA. The shown effect size was clinically relevant and evident even in everyday clinical practice setting, suggesting the potential of including researched genes in the well-established clinical prediction models, moving towards personalized therapy of T2DM. Nevertheless, we found no significant associations between treatment response and *GLP1R* rs10305420 or *SLC5A2* rs9934336.

The observed difference in the reduction of HbA1c between both investigated *GLP1R* polymorphisms is concordant with the previously reported from *in vitro* functional studies which showed lower cell surface expression and cAMP mediated intracellular signaling of the receptor carrying Ser168 characteristic for rs6923761 as compared to Leu7 characteristic for rs10305420 variant (8). In T2DM patients, the normal *GLP1R* rs6923761 G allele was statistically and clinically significantly associated with a greater reduction in HbA1c following the treatment with GLP-1RA than the polymorphic A allele. Similarly, a GWAS study of observational data and large randomized controlled trials by Dawed et al. showed a lower reduction in polymorphic allele carriers (20). However, a trial by de Luis et al. in 90 overweight T2DM patients treated with liraglutide did not show an effect of the rs6923761 polymorphism on HbA1c reduction as we did in our study (21). Although the researchers did not show an effect of the polymorphism, the other trials about dipeptidyl peptidase-4 (DPP-4) inhibitors and their mechanism of action suggest that the rs6923761 polymorphism might nevertheless influence the response to T2DM treatment, as shown in our study. As DPP-4 is an enzyme that degrades incretins, including GLP-1, it is intriguing that some studies showed an effect of the mentioned polymorphism on the glycemic response to DPP-4 inhibitors. A Slovak study of 206 patients with T2DM found that the polymorphic allele was associated with a smaller change in HbA1c after six months of treatment with DPP-4 inhibitors. Similarly, the response to gliptin treatment was also studied by Javorsky et al. who, in a cohort of 140 patients with T2DM, also found a smaller response in HbA1c change with gliptin treatment (22). We hypothesize that the polymorphic GLP-1R might be resistant to both endogenous (as shown with DPP-4 inhibitors) and exogenous (as shown in our study with GLP-1RA) agonists. This assumption is supported by findings from a study by de Luis et al., which revealed that diabetic patients carrying the A allele of the rs6923761 *GLP1R* polymorphism had higher basal GLP-1 levels compared to non-carriers (23). Moreover, a study by Michałowska et al. illustrated that carriers of polymorphic allele of rs6923761 had higher body mass compared to normal allele carriers, and higher glucose concentrations compared to heterozygotes (24). Regardless, the data on this issue is not consistent, highlighting the need for further research to explore the underlying mechanisms behind the differential responses of patients to treatment.

Nevertheless, in our study, the observed difference in HbA1c change between normal and polymorphic allele carriers (more than 1 percentage point) is clinically important, as reductions in HbA1c lower the risk of complications and consequently decrease healthcare costs. A meta-analysis of observational studies by Selvin et al. demonstrated that a 1-percentage point increase in HbA1c is associated with the relative risk of 1.18 (95% confidence intervals (CI): 1.10 to 1.26) for any cardiovascular disease event in patients with T2DM (25). Supporting this, the UKPDS 35 observational study involving 3,642 patients revealed that each 1-percentage point decrease in HbA1c was associated with a 21% (95% CI: 15%–17%) reduction in T2DM-related deaths, a 14% (95% CI: 8%–21%) reduction in myocardial infarctions, and a 37% (95% CI: 33%–41%) reduction in microvascular complications (26). Similarly, the study by Zoungas et al. reported a 38% (95% CI: 30%–47%) higher risk of macrovascular event, a 40% (95% CI: 33%–47%) higher risk of a microvascular event, and a 38% (95% CI: 29%–48%) higher risk of death for every 1-percentage point increase in HbA1c above the threshold of 7% (27). Additionally, the healthcare cost impact of HbA1c reduction was assessed by Lage et al., whose study on USA T2DM patients showed that a 1-percentage point reduction in elevated HbA1c was associated with a 1.7% reduction in all-cause total healthcare costs and a 6.9% reduction in T2DM-related healthcare costs, translating to annual cost savings of $545 and $555, respectively (28).

Nevertheless, we have not observed a statistically significant impact of chosen *GLP1R* polymorphisms on treatment effect regarding blood pressure, or body mass in T2DM patients, receiving GLP-1RA. A study by de Luis et al. in 90 overweight T2DM patients treated with liraglutide showed that patients with the polymorphic rs6923761 allele lost more body and fat mass than patients with the normal allele. Similarly to our study, they did not detect an effect on blood pressure reduction (21). A study by Maselli et al. on 59 obese patients without T2DM showed that the polymorphic allele was statistically significantly associated with a greater reduction in body fat but not in body mass after 16 weeks of treatment with liraglutide (29). The change in body composition could explain the beneficial metabolic effect, independent of total body mass. Additionally, one study found that the rs6923761 polymorphic allele was associated with a greater delay in gastric emptying in patients, treated with liraglutide or exenatide, but did not affect body mass change (30). In brief, although the existing literature provides some insights into the impact of the rs6923761 polymorphism on various outcomes, further investigation is warranted to elucidate its precise role, particularly regarding body mass and body composition changes.

In our study, no statistically significant associations with any of our primary outcome variables were observed for *GLP1R* rs10305420 polymorphism, although some studies have suggested the possibility of an effect of this polymorphism on the response to GLP-1RA treatment. For example, a recent study by Yu et al. in 285 overweight patients with T2DM showed a lower reduction in body mass and HbA1c after 6 months of exenatide treatment in carriers of the polymorphic T allele (31). Similarly, a poorer response to body mass loss in the polymorphic allele was also demonstrated in a study by Jensterle et al. in 57 overweight patients with polycystic ovary syndrome treated with liraglutide for 12 weeks (32). In contrast, another study showed no effect of the rs10305420 genotype on the change in fasting glucose, HbA1c, or body mass in 176 patients with T2DM receiving exenatide or liraglutide for 12 weeks (33), similar to our study. Nevertheless, it is possible that we were not able to observe an effect of the chosen polymorphism due to the smaller sample of patients and shorter follow-up period than in the study done by Yu et al. (31).

In our study, we have not observed any statistically significant association between *SLC5A2* rs9934336 and chosen outcomes following treatment with SGLT2 inhibitors in a dominant model. After an exhaustive literature review, we found only one study investigating the effect of this polymorphism on the response to SGLT2 inhibitor treatment. A cross-sectional study by Zimdahl et al. included 2600 patients from a phase III trial of empagliflozin. The study found no statistical association of change in body fat, insulin response, HbA1c, plasma glucose concentration, systolic blood pressure or body mass with any of the five polymorphisms studied, including rs9934336 polymorphism. Notably, this study differed from ours as it examined the effect of only one SGLT2 inhibitor, empagliflozin, and was conducted retrospectively (16). However, the clinically non-observed potential mechanism underlying the influence of rs9934336 polymorphism on the greater reduction in systolic blood pressure can be elucidated by a study conducted by Monoba et al. They showed that the polymorphic allele was associated with higher urinary glucose excretion with lower mean blood glucose levels (13). This finding suggests that with a normal allele, the therapeutic potential of glucose reabsorption inhibition by SGLT2 inhibitors may be slightly greater. Moreover, the *SLC5A2* polymorphism may have a diminished effect on HbA1c and body mass loss. Research has shown that the SGLT1 carrier partially compensates for the reduced function of SLGT2, thereby potentially blunting the impact of the polymorphism (34, 35). Furthermore, while some studies have not directly examined the effect of the polymorphism on treatment response, they have shown that the polymorphic allele of the selected polymorphism also reduces the risk of T2DM and heart failure, albeit increasing the risk of diabetic retinopathy (13, 36–38). Despite not showing any significant associations in current data, more studies are needed to elucidate the role of this polymorphism in treatment response to different SGLT2 inhibitors.

When considering the overall effect of the antihyperglycemic drugs in our study, the GLP-1RA group exhibited superior treatment outcomes in reducing HbA1c and blood pressure compared to those reported in existing studies and meta-analyses. For instance, Yeh et al. conducted a meta-analysis of 31 double-blind randomized controlled trials involving 22,948 participants, which demonstrated a less pronounced effect of GLP-1RA on HbA1c (39). Similarly, another meta-analysis of 26 trials with 186,565 patients by Ilias et al. reported a lesser impact of these drugs on blood pressure (40). On the other hand, while SGLT2 inhibitors showed a slightly greater reduction in body mass than previously reported, the reduction in blood pressure was less marked compared to the data from meta-analyses of randomized clinical trials (41–44). The enhanced treatment responses are likely attributable to several factors, including the concurrent administration of multiple drugs. Moreover, our patient cohort predominantly consisted of overweight individuals, with the median patient meeting the obesity criteria as defined by the World Health Organization (WHO). Not surprisingly, this is consistent with the well-described role of obesity in the development and progression of T2DM and is also relevant for the treatment effect (45, 46). Notably, it has been shown that GLP-1RAs cause the greatest body mass loss in patients with BMI > 30 kg/m^2^ (47). In addition, mass loss may affect the lowering of HbA1c, and given the high prevalence of dyslipidemia among our patients, it could have a beneficial impact on lipid profiles (48, 49). Nearly all patients in our group also presented with arterial hypertension, potentially amplifying the treatment efficacy of lowering both systolic and diastolic blood pressure in the GLP-1RA group (50).

The primary limitation of our investigation was the relatively modest sample size. We tried to limit this drawback by selecting only polymorphisms, known to be prevalent in European populations based on prior studies. Although the polymorphisms selected in this study do not capture the full genetic variability of the gene, they were chosen based on their high frequency in the population and their known functional impact on the expression or function of the protein. Moreover, we have decided to use the dominant genetic model in data analysis due to the smaller sample size and to increase the study power. Another limitation is the relatively short follow-up duration (3–6 months), which may not capture longer-term treatment effects, particularly with respect to sustained glycemic control and weight trajectory. Our study was not double-blind, potentially impacting the efficacy of treatment with newer antihyperglycemic drugs. Nevertheless, the study was similar in size and methodologically to other pharmacogenomic studies in this respect (21, 33, 51). Furthermore, our sample’s laboratory and clinical characteristics are comparable to those of patients included in other studies exploring the impact of different *GLP1R* and *SLC5A2* polymorphisms on antihyperglycemic treatment (16, 21, 51). However, patients in our study were treated with multiple antihyperglycemic drugs rather than a single agent. Moreover, although all participants were clinically stabilized on background antihyperglycemic medications prior to study entry, they continued on a range of therapies during the follow-up period. This heterogeneity reflects real-world clinical complexity but complicates the attribution of treatment effects to a single pharmacologic agent. Despite this, patients received either GLP-1R agonists or SGLT2 inhibitors in combination with well-defined background regimens, and our analysis focused specifically on the genotype-treatment interaction with the newly introduced drug class. Moreover, measured variables such as diet, physical activity, and psychosocial factors—each of which may influence treatment outcomes—were not systematically collected. Although these limitations constrain causal inference, they also underscore the relevance of our findings to routine diabetes care, where such complexities are inherent. Even so, included T2DM patients represent those typically encountered by diabetologists in their daily practice. Despite being aware of the methodological limitations mentioned, our intention was to reflect real-world clinical practice and depict treatment responses in a naturalistic setting rather than under strictly controlled conditions typically found in clinical trials. Importantly, all patients were extensively clinically characterized, as evidenced in the **Supplementary Material** provided.

## 5 Conclusions

This study sheds light on previously unclear aspects of the role of specific *SLC5A2* and *GLP1R* polymorphisms in response to SGLT2 inhibitor and GLP-1RA therapies in a clinically well-described patient sample from everyday clinical practice. Our findings indicate an association between *GLP1R* rs6923761 and clinically relevant changes in HbA1c following GLP-1RA treatment. These results offer novel insights into how an individual’s genetic background influences their response to novel classes of antihyperglycemic drugs. Such research may lead to a more efficient use of these drugs, sparing patients from unnecessary treatments and alleviating financial burdens on the healthcare system.

## Data Availability

The datasets presented in this study can be found in online repositories. The names of the repository/repositories and accession number(s) can be found in the article/**Supplementary Material**.
