# Supplementary material for: Genetic variability in sodium-glucose cotransporter 2 and glucagon-like peptide 1 receptor effect on glycemic and pressure control in type 2 diabetes patients treated with SGLT2 inhibitors and GLP-1RA in the everyday clinical practice
Source: Front Endocrinol (Lausanne). 2025 Jul 7;16:1547920. doi: 10.3389/fendo.2025.1547920 (PMC12277154; doi:10.3389/fendo.2025.1547920)
Supplement: Supplementary file 1 [file DataSheet1.docx]

Supplementary Material

# Clinical characteristics

Out of 164 enrolled patients, 161 with T2DM were included in the final analysis. Data on primary endpoints are missing for two patients due to missed appointments, and the genotype of one patient could not be determined.

**Supplementary table 1.** Antihyperglycemic drugs received by the subjects (N = 161).

| **GLP-1RA** | **SGLT2 inhibitors** | **bigvanides** | **sulfonylureas** | **insulin** | **DPP-4 inhibitors** | **Other** | **N** |  |
| --- | --- | --- | --- | --- | --- | --- | --- | --- |
|  |  |  |  |  |  |  | **4** | **Number of patients with the described combination of antihyperglycemic drugs** |
|  |  |  |  |  |  |  | **18** |  |
|  |  |  |  |  |  |  | **5** |  |
|  |  |  |  |  |  |  | **1** |  |
|  |  |  |  |  |  |  | **5** |  |
|  |  |  |  |  |  |  | **1** |  |
|  |  |  |  |  |  |  | **7** |  |
|  |  |  |  |  |  |  | **1** |  |
|  |  |  |  |  |  |  | **1** |  |
|  |  |  |  |  |  |  | **10** |  |
|  |  |  |  |  |  |  | **2** |  |
|  |  |  |  |  |  |  | **11** |  |
|  |  |  |  |  |  |  | **4** |  |
|  |  |  |  |  |  |  | **7** |  |
|  |  |  |  |  |  |  | **21** |  |
|  |  |  |  |  |  |  | **24** |  |
|  |  |  |  |  |  |  | **1** |  |
|  |  |  |  |  |  |  | **2** |  |
|  |  |  |  |  |  |  | **5** |  |
|  |  |  |  |  |  |  | **1** |  |
|  |  |  |  |  |  |  | **13** |  |
|  |  |  |  |  |  |  | **17** |  |
| **70** | **124** | **113** | **61** | **66** | **3** | **3** |  | |
| **Number of patients with a medicine in above mentioned class (N)** | | | | | | |  |  |

The box is marked in grey if the patient has received the particular medicine or their combination and in white if they have not. The right-hand column shows the number of patients with the corresponding combination of medicines of the same line, and the last row shows the number of patients treated with that class of medicines. GLP-1RA - glucagon-like peptide 1 receptor agonists, SGLT2 - sodium-glucose co-transporter 2, DPP-4 - dipeptidyl peptidase 4, N - number of patients.

**Supplementary table 2.** Frequency of micro- and macrovascular late complications of T2DM in the study group (N = 161).

| **Type of complication** | **Category** | **N (%)** |
| --- | --- | --- |
| Microvascular complications | No | 116 (72.0) |
|  | Yes | 45 (28.0) |
| Neuropathy | No | 154 (95.7) |
|  | Yes | 7 (4.3) |
| Nephropathy | No | 151 (93.8) |
|  | Yes | 10 (6.2) |
| Retinopathy | No | 126 (78.3) |
|  | Yes | 35 (21.7) |
| Macrovascular complications | No | 140 (87.0) |
|  | Yes | 21 (13.0) |
| Ischemic heart disease | No | 144 (89.4) |
|  | Yes | 17 (10.6) |
| Peripheral arterial disease | No | 156 (96.9) |
|  | Yes | 5 (3.1) |

**Supplementary table 3.** Response to antihyperglycemic treatment in the whole study group.

| **Variable under study** | **Before treatment** | **After treatment** | **p-value^a^** |
| --- | --- | --- | --- |
| HbA1c (%) | 8.4 (7.7-9.2) | 7.4 (6.8-8.2) [1] | **< 0.001** |
| HbA1c (mmol/mol) | 68.3 (60.7-77.0) | 57.4 (50.8-66.1) [1] | **< 0.001** |
| Body mass (kg) | 89 (79.5-108.5) | 86 (76-103) [2] | **< 0.001** |
| Systolic blood pressure (mmHg) | 143 (135-160) | 140 (125.8-151.5) [3] | **< 0.001** |
| Diastolic blood pressure (mmHg) | 81 (75-88) | 80 (74-86.3) [3] | 0.037 |

^a^ Calculated using the Wilcoxon test for related samples. Missing data are presented in square brackets. For one patient we have no data on HbA1c, body mass or blood pressure after treatment, for one on body mass after treatment and for two on blood pressure after treatment. HbA1c - hemoglobin A1c, also glycated hemoglobin. Only patients that had data for a specific variable available in both time points were included in the analysis of that variable.

# Changes in clinical and laboratory parameters after treatment with GLP-1RA

**Supplementary table 4.** Effect of antihyperglycemic drugs in a subgroup of T2DM patients receiving GLP-1RA.

| **Variable under study** | **Before treatment** | **After treatment** | **p-value^a^** |
| --- | --- | --- | --- |
| HbA1c (%) | 8.5 (7.6-9.4) | 7.1 (6.5-8.0) | **< 0.001** |
| HbA1c (mmol/mol) | 69.4 (59.6-79.2) | 54.1 (47.5-63.9) | **< 0.001** |
| Body mass (kg) | 100.5 (86.8-115) | 95.5 (82-110) | **< 0.001** |
| Systolic blood pressure (mmHg) | 147.5 (135-160) | 138 (129.8-150) | **< 0.001** |
| Diastolic blood pressure (mmHg) | 83.5 (77-90.3) | 80 (75-87) | **0.028** |

^a^ Calculated using the Wilcoxon test for related samples. HbA1c - hemoglobin A1c, also glycated hemoglobin.

# Changes in clinical and laboratory parameters after treatment with SGLT2 inhibitors

**Supplementary table 5.** Effect of antihyperglycemic drugs in a subgroup of T2DM patients receiving SGLT2 inhibitors.

| **Variable under study** | **Before treatment** | **After treatment** | **p-value^a^** |
| --- | --- | --- | --- |
| HbA1c (%) | 8.4 (7.7-9.1) | 7.6 (6.9-8.2) [1] | **< 0.001** |
| HbA1c (mmol/mol) | 68.3 (60.7-76.0) | 59.6 (51.9-66.1) [1] | **< 0.001** |
| Body mass (kg) | 87 (78-104) | 83.5 (75-100.3) [2] | **< 0.001** |
| Systolic blood pressure (mmHg) | 140.5 (132.5-157.3) | 140 (125-150) [3] | **0.020** |
| Diastolic blood pressure (mmHg) | 80 (75-86) | 80 (73.5-85) [3] | 0.131 |

^a^ Calculated using the Wilcoxon test for dependent samples. HbA1c - hemoglobin A1c, also glycated hemoglobin. Missing data are presented in square brackets.
